# Characteristics and Predictors of Alzheimer’s Disease Resilience Phenotype

**DOI:** 10.3390/jcm12072463

**Published:** 2023-03-23

**Authors:** Mo-Kyung Sin, Yan Cheng, Jeffrey M. Roseman, Caitlin Latimer, Ali Ahmed, Edward Zamrini

**Affiliations:** 1 College of Nursing, Seattle University, Seattle, WA 98122-1090, USA; 2 The School of Medicine & Health Sciences, George Washington University, Washington, DC 20037, USA; 3 School of Public Health, University of Alabama at Birmingham, Birmingham, AL 35233, USA; 4 Laboratory Medicine & Pathology, University of Washington, Seattle, WA 98104, USA; 5 VA Medical Center, Washington, DC 20242, USA; 6 Georgetown University, Washington, DC 20057, USA; 7 Irvine Clinical Research, Irvine, CA 92614, USA

**Keywords:** Alzheimer’s dementia, blood pressure, resilience

## Abstract

Alzheimer’s disease (AD) is characterized by cognitive impairment in the presence of cerebral amyloid plaques and neurofibrillary tangles. Less is known about the characteristics and predictors of resilience to cognitive impairment in the presence of neuropathological evidence of AD, the focus of this study. Of 3170 adults age ≥65 years in the National Alzheimer’s Coordinating Center (NACC) brain autopsy cohort, 1373 had evidence of CERAD level moderate to frequent neuritic plaque density and Braak stage V–VI neurofibrillary tangles. Resilience was defined by CDR-SOB and CDR-Global scores of 0–2.5 and 0–0.5, respectively, and non-resilience, CDR-SOB and CDR-Global scores >2.5 and >0.5, respectively. Multivariable logistic regression models were used to examine the independent associations of patient characteristics with resilience. There were 62 participants (4.8%) with resilience. Those with resilience were older (mean age, 88.3 vs. 82.4 years), more likely to be women (61.3% vs. 47.3%) and had a lower prevalence of the APOE-e4 carrier (41.9% vs. 56.2%). They also had a higher prevalence of hypertension, heart failure, atrial fibrillation, diuretic use, beta-blocker use, and APOE-e2 carrier status. Greater age at death, diuretic use, and APOE-e2 were the only characteristics independently associated with higher odds of the AD resilience phenotype (adjusted OR, 1.09; 95% CI, 1.05–1.13; *p* < 0.01; 2.00 (1.04–3.87), *p* = 0.04, 2.71 (1.31–5.64), *p* < 0.01, respectively). The phenotype of resilience to cognitive impairment is uncommon in older adults who have neuropathological evidence of AD.

## 1. Introduction

Alzheimer’s disease (AD) is the leading cause of dementia in older adults. Neuropathologic evidence of AD includes the presence of extracellular amyloid β (Aβ) plaques and intracellular neurofibrillary tangles of hyperphosphorylated tau (pTau) [1]. Multiple studies have established the association between AD pathology and clinical symptoms of cognitive impairment [2,3,4,5,6]. Although most people with substantial neuropathological evidence of AD will ultimately develop cognitive impairment or dementia [1], findings from autopsy studies suggest a phenotype of clinical resilience despite neuropathology [7,8]. This is akin to the phenotype of asymptomatic left ventricular systolic dysfunction in which patients do not develop symptoms of heart failure despite a low ejection fraction [9]. A better understanding of this poorly characterized phenotype of resilience may provide insights into maintaining good cognition and prevention of AD. The objective of the current study was to examine the characteristics and predictors of resilience to cognitive decline in older adults with neuropathological evidence of AD. 

## 2. Materials and Methods

### 2.1. Data and Participants

We analyzed the National Alzheimer’s Coordinating Center (NACC) autopsy data collected by the National Institute on Aging (NIA)-funded Alzheimer’s Disease Centers (ADCs). The NACC was established in 1991 by the NIA/NIH (U01 AG016976) to facilitate collaborative AD research. It maintains a large relational database of standardized clinical and neuropathological data, contributed to by the approximately 39 past and present ADCs, where all enrolled participants undergo a standardized evaluation. From 2005 to the present, ADCs have been contributing data to the Uniform Data Set (UDS), collected prospectively by clinicians, neuropsychologists, and other ADC research personnel using up to 18 standardized forms for each visit. The time period covered includes initial UDS visits and approximately annual follow-up visits, plus milestones such as death or dropout. The UDS was expanded with two modules: the FTLD module (detailed clinical information related to frontotemporal lobar degeneration) implemented in 2012 and the LBD module (information on Lewy body disease) implemented in 2015. Data collection for the UDS, including the FTLD and LBD modules, is ongoing. The NACC monitors the reliability of the data via range and logic checks. Written consent was obtained from participants and the study was approved by the institutional review board at each site. 

Of the 3170 participants, 65 years of age or older at death, who underwent autopsy between 2005–2020, 1415 had neuropathological evidence of substantial AD, defined by both the Consortium to Establish a Registry for Alzheimer’s Disease (CERAD) level moderate to frequent neuritic plaque density and by Braak stages V or VI neurofibrillary tangles in the peristriatal and striatal cortex. We used Clinical Dementia Rating (CDR) scores to categorize the study participants into resilient and non-resilient. Resilience was defined by CDR Sum of Boxes (SOB) and CDR-Global scores 0–2.5 and 0–0.5; non-resilience by CDR-SOB and CDR-Global scores of >2.5 and >0.5. We excluded 42 individuals with discordant CDR scores (CDR-SOB >2.5 but CDR-Global 0–0.5, and CDR-SOB 0–2.5 but CDR-Global >0.5). 

### 2.2. AD Pathology

Neuritic plaque density was quantified according to the CERAD level and categorized as none, sparse, moderate, and frequent [10]. Neurofibrillary tangle distribution was assessed using the Braak stage and categorized as stage I (transentorhinal region), stage II (entorhinal region), stage III (temporo-occipital gyrus), stage IV (temporal cortex), stage V (peristriatal cortex), and stage VI (striatal cortex) [11]. 

### 2.3. Cognitive Status 

The CDR-SOB and CDR-Global scores within a year prior to death were used to assess cognitive status [12]. The CDR-SOB is categorized 0 as no cognitive impairment, 0.5–4 as questionable cognitive impairment, 4.5–9 as mild dementia, 9.5–15.5 as moderate dementia, and 16–18 as severe dementia [12]. CDR-Global cognitive functioning measures 6 domains: memory, orientation, judgment and problem solving, community affairs, home and hobbies, and personal care with each domain rated on a 5-point scale of functioning as follows: 0 = no impairment, 0.5 = questionable impairment; 1 = mild impairment, 2 = moderate impairment, and 3 = severe impairment (personal care is scored on a 4-point scale without a 0.5 rating available) [12].

### 2.4. Other Participant Characteristics

Data on demographic and other patient characteristics included age at death, sex, race, ethnicity, education years, APOE-e4 carrier status (0 = absence of the ε4 allele, 1 = 1 or 2 copies of the ε4 allele), APOE-e2 carrier status (0 = absence of the ε2 allele, 1 = 1 or 2 copies of the ε2 allele), cardiovascular risk factors/disease (e.g., smoking history, body mass index, blood pressure, anti-hypertensive medication use, diabetes mellitus, stroke, myocardial infarction, heart failure, atrial fibrillation) and other neuropathologic findings (e.g., Lewy bodies, hippocampal sclerosis, atherosclerosis, cerebral amyloid angiopathy, and cortical and deep cerebral microinfarcts). Data on blood pressure (BP) were collected from all visits prior to death. We used two ways to estimate blood pressures for each patient: BP at the last visit and mean of BP from all visits. Hypertension was defined as systolic BP ≥140 or diastolic BP ≥90 mmHg in at least two visits and/or ever treated with anti-hypertensive agents. Anti-hypertensive agent use was self-reported and measured from all visits prior to death. To keep the sample size as large as possible, we did not exclude the patients with missing values. If a variable had a missing value, we converted the missing value into the category of ‘unknown’.

### 2.5. Statistical Analysis 

We compared patient characteristics between two outcome groups (AD resilient vs. non-resilient participants). Depending on the nature of the variables, we compared the continuous variables between two groups using Student’s *t*-tests and compared categorical variables between the two groups using chi-square tests (or Fisher’s exact tests when more than 20% of the cells had expected frequencies <5).

Two multiple logistic regression models were fit to estimate the independent associations between the patient characteristics and the phenotype of AD resilience: one for the overall population and the other for those with hypertension. The main predictors in the two models included: age at death, sex, race, hypertension (only for the overall population model), beta-blocker use, diuretic use, APOE-e4, APOE-e2, heart failure, and atrial fibrillation. The other covariates adjusted in the models included education, smoking (ever), diabetes mellitus, stroke, myocardial infarction, the use of angiotensin-converting enzyme inhibitors or angiotensin II inhibitors, calcium channel blocking agents, last body mass index (kg/m^2^), and mean systolic and diastolic BP from all visits (mmHg). Multiple comparisons may not be a concern of the study, because the final estimates between the predictors and the outcome were based on a single model from two different cohorts, the overall cohort and subgroup of hypertensive patients, respectively.

Considering the known association between BP and the risk of AD, we compared the observed and predicted BP trajectories between the two groups. SBP trajectories were developed based on the time repeated SBP measures using the SAS^®^ (version 9.4, SAS Institute Inc., Cary, NC, USA) Proc Traj program [13], which provides a curve of average SBP measures (solid curve) and a predicted trajectory (dashed curve). Plots were created for resilient and non-resilient patients, respectively. In each plot, the x-axis indicates years prior to death, with the date of death at the zero point on the x-axis; the y-axis indicates the BP measure. We used all the BP measures within 12 years prior to death. This was because only 18 patients (1.3%) had BP measures more than 12 years prior to death. BP measures were captured at the year level. If a patient had multiple measures at a specific year interval, then we used the measure closest to the end of that interval. To fit a predicted trajectory, the regression models with and without quadratic and/or cubic terms were fitted to identify the model of best fit choosing the one with the lowest Bayesian information criterion (BIC). The beta coefficients and their *p*-values of each model were estimated to evaluate the association between BP and time. All analyses were performed using SAS 9.4. The level of significance was set at <0.05, two-tailed. 

## 3. Results

### 3.1. Participant Characteristics

Specifically, 3170 participants were aged ≥65 years old at death and underwent an autopsy between 2005 and 2020. Of those, 1415 met the neuropathological criteria for advanced AD. Moreover, 62 participants (4.8%) were categorized as resilient, 1311 (95.2%) as non-resilient, and 42 were excluded for discordant CDR scores. The study participants with the AD resilience phenotype died at an older age (mean age, 88.3 vs. 82.4 years), were more often women (61.3% vs. 47.3%), and had a lower prevalence of APOE-e4 (41.9% vs. 56.2%) and a lower proportion of CERAD and Braak VI stage, compared to those without resilience (Table 1). Those with resilience had a higher prevalence of hypertension (83.9% vs. 71.8%; *p* = 0.04), heart failure (12.9% vs. 5.7%, *p* = 0.02), and atrial fibrillation (25.8% vs. 12.3%, *p* < 0.01; Table 1).

### 3.2. Predictors of Resilience to AD

Age at death, diuretic use, and APOE-e2 were the only characteristics independently associated with higher odds of the AD resilience phenotype (adjusted odds ratio with 95% CI: 1.09 (1.05–1.13); 2.00 (1.04–3.87); 2.71 (1.31–5.64), respectively) (Table 2). Odds ratios (95% CIs) associated with the female sex and African American race were 1.32 (0.76–2.31) and 1.22 (0.41–3.66), respectively. The presence of the APOE-e4 gene was associated with lower odds of AD resilience, but not statistically significant (adjusted odds ratio, 0.69; 95% CI, 0.40–1.20). The odds ratios (95% CIs) for AD resilience associated with other patient characteristics are displayed in Table 2. Similar associations were observed in the subgroup of participants with hypertension, except that the odds ratio (95% CI) associated with the African American race in those with hypertension was 0.98 (0.28–3.38). There was no significant interaction between race and hypertension.

### 3.3. Blood Pressure Trajectory and Resilience

Both observed and predicted systolic BP in older adults with resilience was lower 12 years before death, but between years 8 to 9 before death, it increased and was higher than that in those with non-resilience. Two years before death, systolic BP in the resilience group dropped about 5 mmHg and systolic BP in both groups remained similar thereafter, until death. However, none of these differences were statistically significant. Predicted values of diastolic BP were lower in the resilience group about 10 years before death, but they converged over the years, approaching one another before death.

## 4. Discussion

The findings from our study demonstrate that the phenotype of AD resilience is uncommon in older adults with significant neuropathological evidence of AD, and that several characteristics distinguish these individuals from those who are not resilient. We observed that increasing age, diuretic use, and APOE-e2 carrier status were significantly associated with higher odds of AD resilience independent of other variables. Although other characteristics did not have significant association with the AD resilience phenotype, likely due to the lack of power, numerical differences suggest that other characteristics such as the APOE-e4 non-carrier state may be markers of AD resilience. Interestingly, neither hypertension nor BP had significant association with AD resilience. These findings suggest that while rare, AD resilience in older adults with significant AD neuropathology may provide better insights into AD pathophysiology and prevention of further progression of cognitive impairment and dementia.

Despite the lack of power due to the small sample size and the hypothesis-generating nature of our study, we observed that age, diuretic use, and APOE-e2 were the only significant predictors of the AD-resilient phenotype. This is intriguing considering that age is also a major risk factor for AD. Participants with the AD-resilient phenotype in our study had a mean age of 88 years at the time of death. The AD-resilient participants in our study might be expected to be resilient to other age-related comorbidities, however, they had a higher prevalence of age-related comorbidities, such as hypertension, heart failure, and atrial fibrillation. It is also possible that APOE-e2 influenced both the rate of AD development and aging itself. APOE-e2 is known to have a neuroprotective effect [14,15,16] and is also associated with longevity independent of AD [17,18]. Those APOE-e2 carriers lived longer than APOE-e4 carriers (n = 93, 85 ± 8.1 vs. n = 763, 82 ± 7.6, respectively) in our sub-analysis.

Other studies, such as the Ginkgo Evaluation of Memory Study [19] and the Cache County Study [20], also reported a beneficial effect of diuretics on cognitive function in Alzheimer’s disease. Diuretics were associated with a statistically significant reduction of AD risk (aHR 0.61, 95% CI 0.37–0.98), with potassium-sparing diuretics demonstrating significant risk reduction when used alone (aHR 0.09, 95% CI 0.01–0.41) compared to other anti-hypertensives used without potassium-sparing diuretics (aHR 0.76, 95% CI 0.49–1.15) [20]. 

Although individuals in the AD-resilient group were not immune to neuropathological changes consistent with AD, they were less severe as evidenced by lower CERAD and Braak scores. Others found similar findings, but a stronger association of tau pathology with cognition than amyloid pathology [1,21]. It is possible that AD-resilient individuals had developed skills to minimize symptoms and perform well in CDR tests, similar to those with a higher education [22,23]. Older adults with more than 12 years of education may delay the progression of the CDR-SOB score from 0 to 2.5 by more than 3 years than those with a lower education [14]. However, the participants with and without AD-resilience in our study had comparable education. Yet, it is also possible that these individuals were gifted with pragmatic adaptability that allowed them to compensate for the cognitive decline corresponding to cerebral neuropathology. Finally, it is possible that AD-resilient individuals had higher cerebrospinal fluid levels of fibrillogenic soluble 42-amino acid amyloid-beta-peptide (Aβ_42_) leading to lower levels in the brain, which have been shown to be associated with normal cognition, even in the presence of insoluble cerebral amyloid plaques [24].

Studies have examined dissociation between the cerebral neuropathology of AD and the clinical symptoms of AD [7,8]. In one small autopsy study of 50 individuals, four neuropathological differences (e.g., in neuron numbers, synaptic markers, axonal geometry, and glial activation) were observed between those with and without resilience to AD pathology [7]. The findings from an autopsy study of 276 individuals with intermediate or high levels of AD pathology of whom 68 were cognitively resilient to AD pathology, a higher education was a predictor of AD resilience [8]. However, unlike our study, in that study, a higher proportion of individuals with AD resilience had a higher education (college degrees). Differences in sample size, study populations and methodologies may underlie these differences, and highlight the need for future larger studies to understand this unique phenotype that may provide critical insights to the pathophysiology of AD.

### Limitations

As in any observational study, the findings of our study can be biased by unmeasured confounders. The temporality of the association between AD neuropathology and AD symptoms cannot be fully established as the neuropathological data is postmortem. However, neuropathological changes in AD would be expected to precede AD clinical symptom manifestation. The AD clinical symptoms were assessed by CDR from patients or those who knew the patients. Individuals with higher baseline cognitive skills may compensate for early symptoms or overperform in screening tests, such as CDR. 

We defined resilience based on AD pathology (the key feature of AD) and cognitive status (here, with CDR because NACC used CDR as a cognitive status measure). Others defined resilience based on AD pathology and cognitive status measured with the Cognitive Abilities Screening Instrument (CASI) because the Adult Changes in Thought study (ACT) used CASI for cognitive status measurement [8]. The two studies had significantly different resilience rates (4.5% in our study vs. 25% in the ACT). The use of different definitions of resilience by researchers limits generalizability of the study findings.

The association of diuretics probably represents an indication bias, as these drugs are often prescribed for heart failure. The prevalence of heart failure increases with age and its higher prevalence is likely a marker of older age in the resilient group. Diuretics used for heart failure may be a potential explanation for the higher odds of AD resilience in the older age group. We did not conduct a sub-analysis for the type of diuretics used because it was outside the scope of this study. Future studies can pursue this issue.

## 5. Conclusions

Resilience to cognitive impairment in the presence of significant neuropathological evidence of AD is rare and these individuals seem to have outlived their projected life expectancies, despite a high burden of age-related morbidities. There are characteristics that distinguish the phenotype of resilience from that of non-resilience, but only age, diuretic use, and APOE-e2 were independent predictors of AD resilience. Future adequately powered studies may point to potential neuropathological and genetic predictors of the AD resilience phenotype, which may provide important insights into maintaining good cognition and the prevention of AD. 

## Figures and Tables

**Table 1:** Characteristics of 1373 participants in the National Alzheimer’s Coordinating Center (NACC) autopsy data, by resilience to Alzheimer’s disease (AD).

Mean (SD)/n (%)	No Resilience (n = 1311)	Resilience (n = 62)	*p*-Value
Age at death (years)	82.4 (±8.1)	88.3 (±6.6)	**<0.01**
Female	620 (47.3%)	38 (61.3%)	**0.03**
African American	67 (5.1%)	4 (6.4%)	0.64
CERAD level			
Moderate	273 (20.8%)	28 (45.2%)	**<0.01**
Frequent	1038 (79.2%)	34 (54.8%)	
Braak stage			
V	482 (36.8%)	51 (82.3%)	**<0.01**
VI	829 (63.2%)	11 (17.7%)	
APOE-e4	737 (56.2%)	26 (41.9%)	**0.02**
APOE-e2	82 (6.3%)	11 (17.7%)	**<0.01**
Lewy bodies	527 (40.2%)	20 (32.3%)	0.37
Cerebral microinfarcts	267 (20.4%)	13 (21.0%)	0.93
Cerebral amyloid angiopathy			
No or Mild	689 (52.6%)	37 (59.7%)	
Moderate or Severe	599 (45.7%)	23 (37.1%)	0.33
Unknown	23 (1.7%)	2 (3.2%)	
Hippocampal sclerosis			
No	608 (46.4%)	32 (51.6%)	
Yes	118 (9.0%)	2 (3.2%)	0.27
Unknown	585 (44.6%)	28 (45.2%)	
Education (years)	15.2 (±3.2)	15.6 (±3.2)	0.43
Smoking (ever)	507 (38.7%)	25 (40.3%)	0.79
Hypertension	942 (71.8%)	52 (83.9%)	**0.04**
Diabetes mellitus	175 (13.4%)	7 (11.3%)	0.64
Stroke	113 (8.6%)	7 (11.3%)	0.47
Myocardial infarction	112 (8.5%)	8 (12.9%)	0.24
Heart failure	75 (5.7%)	8 (12.9%)	**0.02**
Atrial fibrillation	161 (12.3%)	16 (25.8%)	**<0.01**
Anti-hypertensive agents use			
Any	677 (51.6%)	40 (64.5%)	0.05
Angiotensin-converting enzyme inhibitors	228 (17.4%)	9 (14.5%)	0.56
Angiotensin II inhibitors	92 (7.0%)	8 (12.9%)	0.08
Beta-adrenergic blocking agents	259 (19.8%)	23 (37.1%)	**<0.01**
Calcium channel blocking agents	190 (14.5%)	11 (17.7%)	0.48
Diuretics	185 (14.1%)	20 (32.3%)	**<0.01**
Last body mass index (kg/m^2^)	25.6 (±4.3)	25.4 (±4.3)	0.68
Last blood pressure (mmHg)			
Systolic blood pressure	131 (20)	133 (19)	0.32
Diastolic blood pressure	73 (8)	71 (11)	0.18
Pulse pressure	58 (18)	63 (15)	0.05
Mean blood pressure of all visits (mmHg)			
Systolic blood pressure	133 (16)	134 (13)	0.35
Diastolic blood pressure	73 (8)	72 (8)	0.19
Pulse pressure	59 (14)	63 (12)	0.05

**Table 2:** Predictors of resilience to Alzheimer’s disease (AD) in 1373 participants in the National Alzheimer’s Coordinating Center (NACC) autopsy data with neuropathological evidence of AD.

	Unadjusted Odds Ratio	Adjusted Odds Ratio	*p* for Adjusted Odds Ratio
**All patients (c-statistic for multiple logistic regression: 0.78)**			
Age at death	1.11 (1.07–1.15)	1.09 (1.05–1.13)	**<0.01**
Female sex	1.77 (1.05–2.97)	1.32 (0.76–2.31)	0.32
African American	1.28 (0.45–3.63)	1.22 (0.41–3.66)	0.71
Hypertension	2.04 (1.02–4.05)	1.04 (0.48–2.24)	0.92
Beta-blocker use	2.41 (1.41–4.11)	1.68 (0.90–3.14)	0.10
Diuretic use	2.91 (1.67–5.08)	2.00 (1.04–3.87)	**0.04**
APOE-e4	0.50 (0.29–0.84)	0.69 (0.40–1.20)	0.19
APOE-e2	3.13 (1.57–6.26)	2.71 (1.31–5.64)	**<0.01**
Heart failure	2.44 (1.12–5.32)	0.96 (0.40–2.33)	0.93
Atrial fibrillation	2.48 (1.37–4.49)	1.51 (0.78–2.91)	0.22
**Hypertensive patients only (c-statistic for multiple logistic regression: 0.75)**			
Age at death	1.09 (1.05–1.13)	1.06 (1.02–1.11)	**<0.01**
Female sex	2.09 (1.18–3.72)	1.51 (0.82–2.80)	0.18
African American	1.09 (0.33–3.63)	0.98 (0.28–3.38)	0.98
Beta-blocker use	2.07 (1.18–3.65)	1.63 (0.88–3.03)	0.12
Diuretic use	2.53 (1.42–4.53)	1.95 (1.02–3.75)	0.05
APOE-e4	0.46 (0.26–0.83)	0.60 (0.32–1.11)	0.10
APOE-e2	2.50 (1.12–5.55)	2.21 (0.95–5.12)	0.06
Heart failure	2.34 (1.06–5.17)	1.05 (0.43–2.54)	0.92
Atrial fibrillation	2.16 (1.16–4.03)	1.51 (0.76–2.97)	0.24

Other variables in the model included education, smoking (ever), diabetes mellitus, stroke, myocardial infarction, the use of angiotensin-converting enzyme inhibitors or angiotensin II inhibitors, calcium channel blocking agents, last body mass index (kg/m^2^), and mean systolic and diastolic blood pressure from all visits (mmHg).

## Data Availability

Data can be obtained through the National Alzheimer’s Coordinating Center at http://www.alz.washington.edu/ (accessed on 3 February 2023). NACC data are contributed by the NIA-funded ADRCs: P30 AG062429 (PI James Brewer, MD, PhD), P30 AG066468 (PI Oscar Lopez, MD), P30 AG062421 (PI Bradley Hyman, MD, PhD), P30 AG066509 (PI Thomas Grabowski, MD), P30 AG066514 (PI Mary Sano, PhD), P30 AG066530 (PI Helena Chui, MD), P30 AG066507 (PI Marilyn Albert, PhD), P30 AG066444 (PI John Morris, MD), P30 AG066518 (PI Jeffrey Kaye, MD), P30 AG066512 (PI Thomas Wisniewski, MD), P30 AG066462 (PI Scott Small, MD), P30 AG072979 (PI David Wolk, MD), P30 AG072972 (PI Charles DeCarli, MD), P30 AG072976 (PI Andrew Saykin, PsyD), P30 AG072975 (PI David Bennett, MD), P30 AG072978 (PI Neil Kowall, MD), P30 AG072977 (PI Robert Vassar, PhD), P30 AG066519 (PI Frank LaFerla, PhD), P30 AG062677 (PI Ronald Petersen, MD, PhD), P30 AG079280 (PI Eric Reiman, MD), P30 AG062422 (PI Gil Rabinovici, MD), P30 AG066511 (PI Allan Levey, MD, PhD), P30 AG072946 (PI Linda Van Eldik, PhD), P30 AG062715 (PI Sanjay Asthana, MD, FRCP), P30 AG072973 (PI Russell Swerdlow, MD), P30 AG066506 (PI Todd Golde, MD, PhD), P30 AG066508 (PI Stephen Strittmatter, MD, PhD), P30 AG066515 (PI Victor Henderson, MD, MS), P30 AG072947 (PI Suzanne Craft, PhD), P30 AG072931 (PI Henry Paulson, MD, PhD), P30 AG066546 (PI Sudha Seshadri, MD), P20 AG068024 (PI Erik Roberson, MD, PhD), P20 AG068053 (PI Justin Miller, PhD), P20 AG068077 (PI Gary Rosenberg, MD), P20 AG068082 (PI Angela Jefferson, PhD), P30 AG072958 (PI Heather Whitson, MD), P30 AG072959 (PI James Leverenz, MD).
